# Comparisons of corneal biomechanical and tomographic parameters among thin normal cornea, forme fruste keratoconus, and mild keratoconus

**DOI:** 10.1186/s40662-021-00266-y

**Published:** 2021-11-16

**Authors:** Lei Tian, Di Zhang, Lili Guo, Xiao Qin, Hui Zhang, Haixia Zhang, Ying Jie, Lin Li

**Affiliations:** 1 Beijing Institute of Ophthalmology, Beijing Tongren Eye Center, Beijing Ophthalmology & Visual Sciences Key Laboratory, Beijing Tongren Hospital, Capital Medical University, Beijing, 100730 China; 2 Beijing Key Laboratory of Fundamental Research on Biomechanics in Clinical Application, Capital Medical University, Beijing, 100069 China; 3 School of Biomedical Engineering, Capital Medical University, Beijing, 100069 China; 4 Beijing Advanced Innovation Center for Big Data-Based Precision Medicine, Beihang University & Capital Medical University, Beijing Tongren Hospital, Beijing, 100730 China

**Keywords:** Thin normal cornea, Forme fruste keratoconus, Mild keratoconus, Corneal biomechanical parameters

## Abstract

**Background:**

To compare the dynamic corneal response (DCR) and tomographic parameters of thin normal cornea (TNC) with thinnest corneal thickness (TCT) (≤ 500 µm), forme fruste keratoconus (FFKC) and mild keratoconus (MKC) had their central corneal thickness (CCT) matched by Scheimpflug imaging (Pentacam) and corneal visualization Scheimpflug technology (Corvis ST).

**Methods:**

CCT were matched in 50 eyes with FFKC, 50 eyes with MKC, and 53 TNC eyes with TCT ≤ 500 µm. The differences in DCR and tomographic parameters among the three groups were compared. The receiver operating characteristic (ROC) curve was used to analyze the diagnostic significance of these parameters. Back propagation (BP) neural network was used to establish the keratoconus diagnosis model.

**Results:**

Fifty CCT-matched FFKC eyes, 50 MKC eyes and 50 TNC eyes were included. The age and biomechanically corrected intraocular pressure (bIOP) did not differ significantly among the three groups (all *P* > 0.05). The index of height asymmetry (IHA) and height decentration (IHD) differed significantly among the three groups (all *P* < 0.05). IHD also had sufficient strength (area under the ROC curves (AUC) > 0.80) to differentiate FFKC and MKC from TNC eyes. Partial DCR parameters showed significant differences between the MKC and TNC groups, and the deflection amplitude of the first applanation (A1DA) showed a good potential to differentiate (AUC > 0.70) FFKC and MKC from TNC eyes. Diagnosis model by BP neural network showed an accurate diagnostic efficiency of about 91%.

**Conclusions:**

The majority of the tomographic and DCR parameters differed among the three groups. The IHD and partial DCR parameters assessed by Corvis ST distinguished FFKC and MKC from TNC when controlled for CCT.

## Background

Keratoconus (KC) is a corneal ectatic disease, which results in progressive thinning and protrusion of the cornea into a conical shape [1]. The structure of collagen fibers changes and the number of collagen fiber layers decreases in KC [2, 3]. In fact, the corneal microstructure already shows changes in the early stages of KC [4]. A series of changes in the corneal microstructure can alter corneal biomechanics. These biomechanical differences might be detected before the changes in the shape or clinical symptoms of KC and are critical for the diagnosis of other eye diseases [5], the selection of refractive surgery [6], and the screening before refractive surgery [7].

Corneal visualization Scheimpflug technology (Corvis ST) applies constant airflow to the corneal central area using a high-speed Scheimpflug camera to obtain corneal biomechanical response. Corvis ST is widely used in the diagnosis of KC [8] and glaucoma [5]. However, the lack of standardization of existing indicators of Corvis ST limits its application in clinical practice [9]. Most of the parameters from the Corvis ST are affected by corneal thickness [10]; for example, central concave curvature at highest concavity and velocity of the second applanation are positively correlated with central corneal thickness (CCT) [11]. However, patients with KC may have a thick cornea, while normal eyes may have a thin cornea, and CCT affects the diagnosis of related corneal diseases through corneal biomechanical parameters. Some studies have demonstrated a difference in corneal biomechanical parameter values between KC and normal eyes [12]. When the normal cornea is thin, the difference in the biomechanical parameters between the KC and normal cornea may change, thereby affecting KC diagnosis.

In this study, we aimed to explore the changes in the dynamic corneal response (DCR) and corneal tomographic parameters in a population of eyes with CCT-matched thin normal cornea (TNC) controls, forme fruste keratoconus (FFKC), and mild keratoconus (MKC) with TCT ≤ 500 µm. This will provide a basis for further understanding of the test results produced by Corvis ST, which would aid in distinguishing KC from TNC when the cornea is thin.

## Methods

### Subjects

This retrospective study included 153 eyes of 153 individuals (thinnest corneal thickness (TCT) range: 440–500 µm [13]), who were divided into three groups: the TNC group included 53 healthy eyes in 53 subjects, FFKC group included 50 FFKC eyes in 50 patients, and MKC group included 50 MKC eyes in 50 patients. The TCT was minimal pachymetry obtained by Scheimpflug imaging (Pentacam). KC eyes were classified according to Pentacam grading topographical KC classification (TKC). TKC grade 1, 1–2, or 2 were grouped as MKC [12, 14, 15]. All participants chose one eye for analysis, i.e., patients who were diagnosed as FFKC, MKC, and in the case of participants with MKC in both eyes and for healthy subjects, one eye was selected randomly.

All patients diagnosed with KC in the cornea clinic of the Beijing Tongren Hospital, from January 2013 to December 2019, were eligible for inclusion in this study. Clinical KC was diagnosed if the eye met the following criteria [16, 17]: (1) an irregular cornea as determined by distorted keratometry mires, distortion of the retinoscopic or ophthalmoscopic red reflex (or a combination of the two); (2) at least one of the following biomicroscopic signs, Vogt’s striae, Fleischer’s ring of 2 mm arc, or corneal scarring consistent with KC. An eye was diagnosed with FFKC if it was the fellow eye of a patient with KC and showed the following features [18, 19]: (1) a normal-appearing cornea on slit-lamp examination, retinoscopy, and ophthalmoscopy; (2) normal topography with no asymmetric bowtie and no focal or inferior steepening pattern; (3) patient had no history of contact lens use, ocular surgery, or trauma. Subjects that had ocular pathology other than KC, history of corneal or ocular surgery, or systemic diseases that might affect the eye were excluded from this study. All subjects had abandoned soft contact lenses or rigid contact lenses at least 1 month before the examination, the intraocular pressure (IOP) range was 10–21 mmHg; it was found that biomechanically corrected IOP (bIOP) is close to the real intraocular pressure [20–22], and thus the value of bIOP was used as IOP in this study.

The institutional review board of the Beijing Tongren Hospital, Beijing, China approved this study, and all participants signed an informed consent form in accordance with the tenets of the Declaration of Helsinki.

### Ocular examination

A comprehensive ocular examination was performed on the eyes of all subjects, including a detailed assessment of uncorrected distance visual acuity, slit-lamp microscopy, fundus examination, tomographic measurements using Pentacam (Oculus; Optikgeräte GmbH, Wetzlar, Germany), and biomechanical examination using the Corvis ST (Oculus; Optikgeräte GmbH, Wetzlar, Germany). All measurements were taken between 09:00 and 17:00 on the same day and by the same trained ophthalmologists. The Pentacam (software version 1.20r134) reconstructed a three-dimensional image of the entire anterior segment of the eye from the anterior surface of the cornea to the posterior surface of the lens utilizing the high-speed rotating Scheimpflug system. The Corvis ST (software version 1.5r1902) evaluated the dynamic corneal deformation in response to an air-puff pulse. The details and principles of the Pentacam and Corvis ST are described elsewhere [23, 24]. Only the scans that the Pentacam and Corvis ST determined as “OK” for their “quality specification” (QS) function were included for subsequent analysis.

**Fig. 1:**
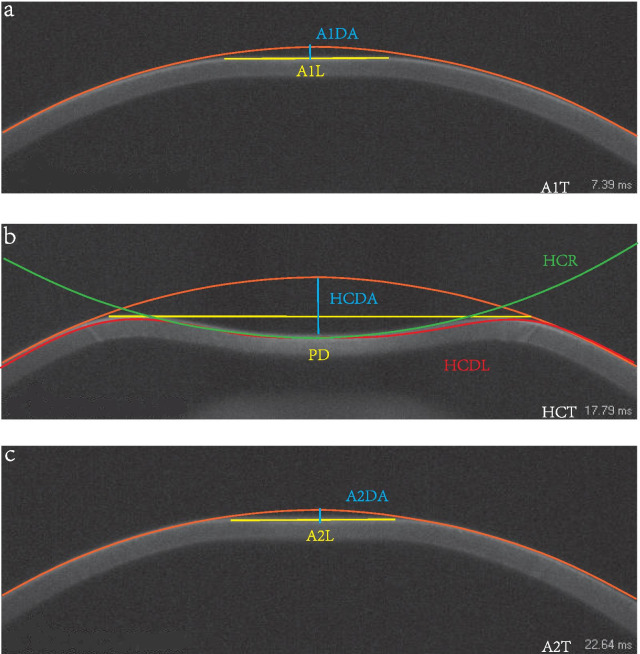
Corvis ST parameters measured, first applanation (**a**), highest concavity (**b**), second applanation (**c**). A1DA, corneal deflection amplitude during the first applanation; A1L, length at the first applanation; A1T, time from starting until the first applanation; HCDA, corneal deflection amplitude at the moment of the highest corneal concavity; PD, peak distance at the highest concavity; HCR, central concave curvature at highest concavity; HCDL, highest concavity deflection length; HCT, time from the measurement beginning to the moment of reaching the highest concavity; A2DA, corneal deflection amplitude during the second applanation; A2L, length at the second applanation; A2T, time from starting until the second applanation

The Corvis ST output parameters mainly included—First applanation: time from starting until the first applanation (A1T), velocity of the corneal apex during the first applanation (A1V), corneal deflection amplitude during the first applanation (A1DA), length at the first applanation (A1L) (Fig. 1a); Second applanation: time from starting until the second applanation (A2T), velocity of the corneal apex during the second applanation (A2V), corneal deflection amplitude during the second applanation (A2DA), length at the second applanation (A2L) (Fig. 1c); Highest concavity: time from the measurement beginning to the moment of reaching the highest concavity (HCT), corneal deflection amplitude at the moment of the highest corneal concavity (HCDA), highest concavity deflection length (HCDL), peak distance at the highest concavity (PD), central concave curvature at highest concavity (HCR), maximum deformation amplitude (DA) (Fig. 1b); other parameters included Ambrósio relational thickness to the horizontal profile (ARTh), deflection amplitude ratio maximal (1 mm and 2 mm) (DAR1, DAR2), stiffness parameter at the first applanation (SP-A1), corneal biomechanical index (CBI), and biomechanically corrected IOP (bIOP). The Pentacam parameters included in the analysis are shown in Table 1.

**Table 1:** Tomographic parameters derived from Pentacam

Parameters		Means
KmF		Mean keratometry from the anterior corneal surface
Kmax		Maximum keratometry from the anterior corneal surface
Astig F		Central astigmatism from the anterior corneal surface
ISV		Index of surface variance
IVA		Index of vertical asymmetry
KI		Keratoconus index
CKI		Central keratoconus index
IHA		Index of height asymmetry
IHD		Index of height decentration

### Statistical analysis

All analyses were performed using SPSS (version 23.0, IBM Corporation, Armonk, NY), MedCalc software (version 19.1, MedCalc Ltd, Ostend, Belgium) and R (version 3.6.3, R Core Team). The CCT was matched between the three groups using propensity score matching. Data were evaluated for normality using Shapiro-Wilk test, and when the data was normally distributed, mean ± SD was used to describe data; whilst the data had a non-normal distribution, the median (range of variation) was used to describe the data. The differences between data were evaluated using one-way ANOVA (multiple comparisons between groups were performed using Bonferroni test) or Kruskal-Wallis test (multiple comparisons between groups were performed using Mann-Whitney U test) was used for non-normal data, and gender differences among the three groups were analyzed using the chi-square test. Power of the tests was calculated using the A1DA data among the three groups. A receiver operating characteristic (ROC) curve analysis was constructed to identify the overall predictive accuracy of parameters and analyze the sensitivity and specificity of these parameters. A *P* value of less than 0.05 was considered statistically significant.

Based on results of comparison among the three groups, we combined DCR and corneal tomographic parameters to establish the keratoconus diagnosis model with back propagation (BP) neural network (MATLAB, R2020b, MathWorks, USA). Randomly selected data (70%) was used as the training set, and the rest of the data was used as the verification set. After testing, the three-layer neural network was selected, the number of neurons in each layer was 5, 3 and 1, trainlm was selected as the activation function. Trainlm is a network training function which is suitable for medium-sized networks and have the fastest convergence speed, the learning rate was set to 0.01, target error was set to 0.005, and the maximum number of iterations was set to 1000 times.

## Results

Table 2 shows the baseline information of eyes categorized by the group with CCT-matched. Kruskal-Wallis test showed that no statistically significant differences were detected for age, CCT, bIOP, among the three groups (all *P* > 0.05); and chi-square test showed that there were no statistically significant differences detected for gender, among the three groups (*P* > 0.05). Furthermore, power of the tests calculated by using the data of A1DA was about 0.99.

**Table 2:** Baseline information of eyes by group with CCT matched

Parameters	TNC (N = 50)	FFKC (N = 50)	MKC (N = 50)	Statistics	*P*
Gender (male/female)	26/24	24/26	27/23	0.374	0.830
Age (years)	24 (17–28)	22 (16–36)	24 (17–32)	2.212	0.331
CCT (μm)	495 (470–507)	495 ± 14	490 ± 14	3.604	0.165
TCT (μm)	492 (467–499)	491 (459–500)	481 (454–500)^#^	6.535	0.038
bIOP (mmHg)	14.1 ± 2.1	14.7 (11.3–20.4)	14.2 (10.8–20.7)	3.135	0.209

*TNC* thin normal cornea; *FFKC* forme fruste keratoconus; *MKC* mild keratoconus; *CCT* central corneal thickness; *TCT* thinnest corneal thickness; *bIOP* biomechanically corrected intraocular pressure

*P* value is for differences between the three groups, ^#^represents statistically significant difference with TNC and MKC

### Changes in the corneal tomographic parameters

**Fig. 2:**
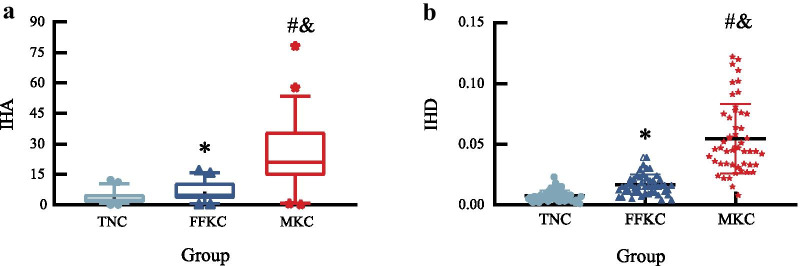
Differences between the three groups with respect to IHA (**a**) and IHD (**b**). *, ^#^ and ^&^represent statistically significant differences between TNC and FFKC, TNC and MKC, and FFKC and MKC, respectively. IHA, index of height asymmetry; IHD, index of height decentration; TNC, thin normal cornea; FFKC, forme fruste keratoconus; MKC, mild keratoconus

The comparison of Pentacam parameters among the three groups are shown in Table 3. Kruskal-Wallis analysis showed that IHA and IHD differed significantly among the three groups (all *P* < 0.001). Except for keratometry from the anterior corneal surface (KmF) and central astigmatism from the anterior corneal surface (Astig F), other tomographic parameters differed significantly between the TNC and MKC groups, FFKC and MKC groups; IHA and IHD were the smallest in TNC groups and the largest in MKC groups (Fig. 2).

**Table 3:** Tomographic parameters of eyes by group

Parameters	TNC	FFKC	MKC	Statistics	*P*
KmF (D)	43.9 ± 1.4	43.7 ± 1.5	44.3 (41.5–48.5)	3.916	0.141
Kmax (D)	45.1 ± 1.7	45.2 ± 1.9	49.0 (43.8–57.3)^#&^	45.110	< 0.001
Astig F (D)	1.1 (0.1–4.6)	1.1 (0.1–2.2)	1.5 (0.1–5.2)	2.806	0.246
ISV	18.68 ± 5.83	19.72 ± 5.66	41.00 (17.00–82.00)^#&^	92.504	< 0.001
IVA	0.11 (0.05–0.35)	0.16 (0.06–0.33)	0.40 (0.16–1.02)^#&^	90.624	< 0.001
KI	1.03 (0.95–1.07)	1.04 (1.00–1.10)	1.10 (1.00–1.23)^#&^	76.787	< 0.001
CKI	1.01 (1.00–1.02)	1.01 (1.00–1.04)	1.02 (0.97–1.13)^#&^	28.187	< 0.001

*TNC* thin normal cornea; *FFKC* forme fruste keratoconus; *MKC* mild keratoconus; *KmF* mean keratometry from the anterior corneal surface; *Kmax* maximum keratometry from the anterior corneal surface; *Astig F* central astigmatism from the anterior corneal surface; *ISV* index of surface variance; *IVA* index of vertical asymmetry; *KI* keratoconus index, *CKI* central keratoconus index

*P* is the value among the three groups. *, ^#^ and ^&^represent statistically significant difference with TNC and FFKC, TNC and MKC, FFKC and MKC, respectively

### Parameters obtained by Corvis ST

The DCR parameters obtained by Corvis ST are shown in Table 4. Eight of 19 and 11 of 19 DCR parameters were significantly different between the TNC and FFKC groups (all *P* < 0.05), and TNC and MKC groups (all *P* < 0.05), respectively. Also, a significant difference was detected for A1DA, A1L, HCDA, HCR, ARTh, and CBI in the FFKC and MKC groups (all *P* < 0.05).

**Table 4:** Corvis ST parameters of eyes by group

Parameters	TNC	FFKC	MKC	Statistics	*P*
A1T (ms)	7.205 ± 0.234	7.121 (6.748–7.801)	7.065 (6.692–7.867)^#^	9.168	0.010^b^
A1V (m/s)	0.153 (0.083–0.194)	0.163 (0.095–0.196)^*^	0.164 ± 0.022^#^	17.483	< 0.001^b^
A1DA (mm)	0.090 ± 0.007	0.097 (0.080–0.142)^*^	0.106 (0.080–0.152)^#&^	51.272	< 0.001^b^
A1L (mm)	2.176 ± 0.147	2.272 ± 0.096^*^	2.335 ± 1.223^#&^	32.121	< 0.001^b^
A2T (ms)	21.873 ± 0.382	22.015 ± 0.371^*^	22.016 (21.004–22.647)^#^	8.612	0.013^b^
A2V (m/s)	− 0.285 ± 0.032	− 0.290 ± 0.039	− 0.305 ± 0.039^#^	3.984	0.021^a^
A2DA (mm)	0.096 (0.083–0.132)	0.107 (0.087–0.548)^*^	0.107 (0.086–0.138)^#^	27.666	< 0.001^b^
A2L (mm)	2.410 (1.525–4.009)	2.579 (1.589–4.510)	2.600 (11.850–4.462)	4.592	0.101^b^
HCT (ms)	16.812 ± 0.441	16.854 ± 0.588	16.969 (15.634–17.855)	3.237	0.198 ^b^
HCDA (mm)	0.957 ± 0.096	1.001 ± 0.128	1.137 ± 0.124^#&^	10.759	< 0.001^a^
HCDL (mm)	6.556 ± 0.393	6.753 (5.250–7.459)	6.657 (5.505–7.323)	3.895	0.143 ^b^
PD (mm)	5.174 (4.665–5.642)	5.247 (4.452–5.719)	5.229 (4.365–5.642)	6.244	0.044 ^b^
HCR (mm)	6.544 ± 0.561	6.668 ± 0.581	6.3260 ± 0.745^&^	3.717	0.027 ^a^
DA (mm)	1.083 ± 0.090	1.137 ± 0.124^*^	1.147 ± 0.095^#^	11.644	0.003 ^b^
ARTh	369.149 (259.978–625.851)	424.172 ± 100.613	313.693 (171.693–641.209)^#&^	23.079	< 0.001^b^
DAR1	1.645 (1.572–1.793)	1.604 (1.507–1.770)^*^	1.630 ± 0.043	15.430	< 0.001^b^
DAR2	4.829 ± 0.340	4.863 ± 0.405	5.094 ± 0.541^#^	7.933	0.019 ^b^
SP-A1	75.114 ± 11.054	73.638 (59.835–109.124)	69.976 (46.205–114.207)	6.846	0.033 ^b^
CBI	0.915 (0.569–0.991)	0.742 (0.000–1.000)^*^	0.964 (0.000–1.000)^&^	22.490	< 0.001^b^

*TNC* thin normal cornea; *FFKC* forme fruste keratoconus; *MKC* mild keratoconus; *A1T* time from starting until the first applanation; *A1V* velocity of the corneal apex during the first applanation; *A1DA* corneal deflection amplitude during the first applanation; *A1L* length at the first applanation; *A2T* time from starting until the second applanation; *A2V* velocity of the corneal apex during the second applanation; *A2DA* corneal deflection amplitude during the second applanation; *A2L* length at the second applanation; *HCT* time from the measurement beginning to the moment of reaching the highest concavity; *HCDA* corneal deflection amplitude at the moment of the highest corneal concavity; *HCDL* highest concavity deflection length; *PD* peak distance at the highest concavity; *HCR* central concave curvature at highest concavity; *DA* maximum deformation amplitude; *ARTh* ambrósio relational thickness to the horizontal profile; *DAR1* the ratio between the deformation amplitude at the apex and at 1 mm from corneal apex; *DAR2* the ratio between the deformation amplitude at the apex and at 2 mm from corneal apex; *SP-A1* stiffness parameter at the first applanation; *CBI* corneal biomechanical index

*P* is the value among the three groups. *, ^#^ and ^&^represent statistically significant difference with TNC and FFKC, TNC and MKC, FFKC and MKC, respectively; ^a^represents one-way ANOVA, ^b^represents Kruskal-Wallis test

### ROC curve analysis

Table 5 shows the data from the FFKC and MKC groups compared to the TNC group for the ROC curve analysis, area under the curve (AUC), sensitivity, specificity, Youden index, and cutoff points for each parameter. A total of seven parameters had sufficient strength (AUC > 0.80) to differentiate MKC from TNC eyes. However, the overall predictive accuracy of these readings except IHD was moderate or inferior for eyes with FFKC (AUC < 0.80), and most of the parameters failed (AUC < 0.70) to differentiate between FFKC and TNC corneas.

**Table 5:** The receiver operating characteristic (ROC) curve analysis values

Parameters		FFKC *vs*. TNC					MKC *vs*. TNC			
	AUC	Sensitivity (%)	Specificity (%)	Youden index	Cutoff	AUC	Sensitivity (%)	Specificity (%)	Youden index	Cutoff
Kmax	0.511	58.00	52.00	0.100	> 45.0	0.840	66.00	90.00	0.560	> 47.0
ISV	0.571	54.00	62.00	0.160	> 19.00	0.980	96.00	96.00	0.920	> 27.00
IVA	0.680	66.00	64.00	0.300	> 0.13	0.976	92.00	92.00	0.840	> 0.21
KI	0.642	64.00	60.00	0.240	> 1.03	0.953	92.00	86.00	0.780	> 1.05
CKI	0.556	34.00	82.00	0.160	≤ 1.00	0.742	64.00	92.00	0.560	> 1.01
IHA	0.722	78.00	66.00	0.440	> 3.0	0.910	80.00	100.00	0.800	> 12.3
IHD	0.849	84.00	72.00	0.560	> 0.008	0.991	96.00	98.00	0.940	> 0.018
A1T	0.594	74.00	48.00	0.220	≤ 7.225	0.673	84.00	48.00	0.320	≤ 7.226
A1V	0.719	58.00	80.00	0.380	> 0.160	0.700	58.00	82.00	0.400	> 0.161
A1DA	0.750	84.00	60.00	0.440	> 0.091	0.880	70.00	92.00	0.620	> 0.098
A1L	0.713	68.00	70.00	0.380	> 2.242	0.795	76.00	78.00	0.540	> 2.253
A2T	0.634	64.00	64.00	0.280	> 21.966	0.660	76.00	56.00	0.320	> 21.918
A2DA	0.766	74.00	72.00	0.760	> 0.101	0.762	62.00	82.00	0.440	> 0.104
HCDA	0.604	56.00	68.00	0.240	> 0.982	0.780	80.00	68.00	0.480	> 0.982
HCR	0.570	42.00	78.00	0.200	> 6.860	0.598	24.00	96.00	0.200	≤ 5.745
DA	0.648	54.00	78.00	0.320	> 1.144	0.691	44.00	88.00	0.320	> 1.163
ARTh	0.616	62.00	64.00	0.260	> 384.769	0.686	38.00	96.00	0.340	≤ 280.029
CBI	0.710	60.00	88.00	0.480	≤ 0.766	0.624	46.00	88.00	0.340	> 0.968

*TNC* thin normal cornea; *FFKC* forme fruste keratoconus; *MKC* mild keratoconus; *AUC* area under the curve; *Kmax* maximum keratometry from the anterior corneal surface; *ISV* index of surface variance; *IVA* index of vertical asymmetry; *KI* keratoconus index; *CKI* central keratoconus index; *IHA* index of height asymmetry; *IHD* index of height decentration; *A1T* time from starting until the first applanation; *A1V* velocity of the corneal apex during the first applanation; *A1DA* corneal deflection amplitude during the first applanation; *A1L* length at the first applanation; *A2T* time from starting until the second applanation; *A2DA* corneal deflection amplitude during the second applanation; *HCDA* corneal deflection amplitude at the moment of the highest corneal concavity; *HCR* central concave curvature at highest concavity; *DA* maximum deformation amplitude; *ARTh* ambrósio relational thickness to the horizontal profile; *CBI* corneal biomechanical index

**Fig. 3:**
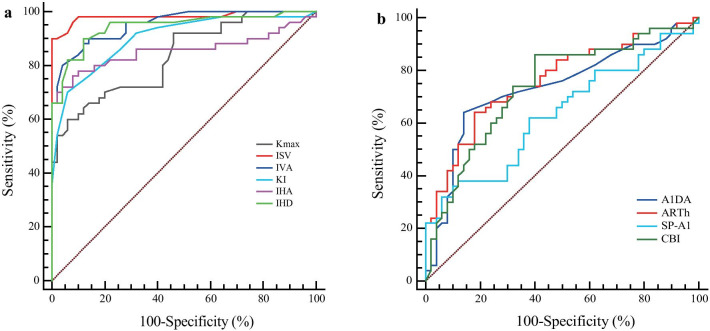
The receiver operating characteristic curves for MKC *vs*. FFKC for Pentacam (**a**) and Corvis ST (**b**) parameters. FFKC, forme fruste keratoconus; MKC, mild keratoconus

The AUC values of some parameters (A1V, A1DA, A1L, A2T, A2DA, DA, DAR1, and CBI) in the FFKC and TNC groups were between 0.634 and 0.766. The AUC values of parameters with statistical differences between MKC and TNC groups were 0.653 to 0.880, and the AUC of A1DA was > 0.80. Kmax, ISV (index of surface variance), IVA (index of vertical asymmetry), KI (keratoconus index), IHA, and IHD had sufficient strength (AUC range: 0.83 to 0.981) to differentiate between the MKC and FFKC eyes (Fig. 3a). Furthermore, ARTh and CBI detected by Corvis ST exhibited moderate strength (AUC = 0.762, cutoff ≤ 338.03; AUC = 0.738, cutoff > 0.766) to differentiate between the MKC and FFKC eyes (Fig. 3b). Also, IHD had sufficient strength to differentiate MKC (AUC = 0.999, cutoff > 0.018) and FFKC (AUC = 0.846, cutoff > 0.010) from TNC eyes, and the MKC from FFKC eyes (AUC = 0.944, sensitivity = 90.00%, specificity = 88.00%, cutoff > 0.025).

**Fig. 4:**
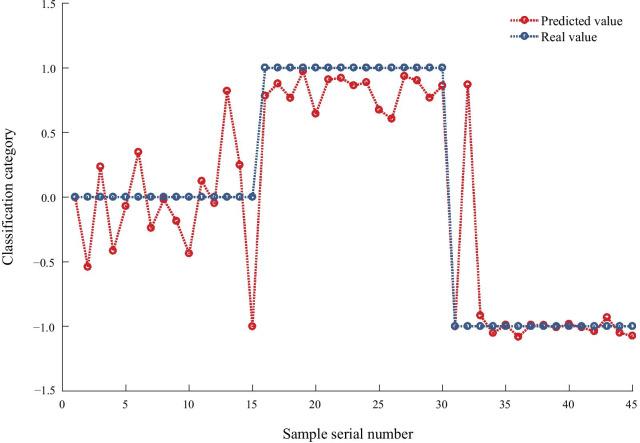
Results of keratoconus (KC) diagnosis with the keratoconus diagnosis model. <  − 0.5: MKC group; [− 0.5, 0.5): FFKC group; ≥ 0.5: TNC group. TNC, thin normal cornea; FFKC, forme fruste keratoconus; MKC, mild keratoconus

According to one-way ANOVA or the Kruskal-Wallis test, 13 parameters (IVA, KI, IHA, IHD, A1V, A1DA, A1L, A2DA, HCDA, DA, ARTh, DAR1, CBI) were used to establish the keratoconus diagnosis model. Figure 4 shows the results with an accuracy of 91%, and the sensitivity (true positive rate) and specificity (true negative rate) of distinguishing FFKC from TNC are 80 and 100%, respectively; the sensitivity and specificity of distinguishing MKC from TNC are 93.3 and 100%, respectively.

## Discussion

Progressive thinning of the cornea is critical for KC development. Intriguingly, thin corneas are prone to corneal ectasia, and the thickness of the cornea is considered while planning the refractive surgery and during the diagnosis of KC. The change in corneal biomechanical properties is considered to be the influencing factor of KC [25], which might be valuable in the early diagnosis of KC than corneal topography. Due to age, IOP and corneal thickness affect the DCR parameters [11, 26], which also affect the application of DCR parameters to diagnose diseases. Therefore, in this study based on the data of Pentacam and Corvis ST, we compared and analyzed the corneal biomechanical properties among TNC, FFKC, and MKC groups, when the CCT of the three groups was matched. The innovation of this study compared with others, is exploring the sensitivity indices for screening keratoconus in thin corneas. The results showed that when CCT among TNC, FFKC, and MKC were matched, IHA, IHD, A1DA, and A1L were different between TNC and FFKC, TNC and MKC, FFKC and MKC. IHD and A1DA parameters from Corvis ST showed that FFKC and MKC could be distinguished from TNC. This suggests that when CCT is thin, IHD and A1DA may have great potential to distinguish FFKC from TNC, and MKC from TNC.

In our study, nine parameters from Pentacam were compared among the three groups. IHD had sufficient (AUC > 0.80) strength and sensitivity to differentiate FFKC and MKC from the TNC, and MKC from FFKC eyes. Schlegel et al. [27] also demonstrated that the anterior surface abnormality of KC often appeared earlier than those for visual acuity and thickness, and IHD showed an optimal discriminating power between mild KC from thin corneas [14]. Our results show that when IHD is more than 0.008, patients should be closely observed, and when IHD is more than 0.018, it indicates an increased risk of KC.

The results of this study showed that the AUCs of A1DA and A2DA were higher between FFKC and TNC groups with A1DA, A1L, and HCDA being able to distinguish between MKC from TNC. This phenomenon designated a good distinguishing potential of A1DA to differentiate FFKC and MKC from TNC eyes. Research has found that the topographic parameters of keratoconus patients are related to DCR parameters [28]. Catalán-López et al. [29] found that the linear combination of A2L and CCT was helpful in discriminating subclinical KC and normal corneas. The above research shows that biomechanics has potential value in the early diagnosis of KC. Previous studies demonstrated that the microstructure [2–4, 30] and the density of endothelial cells [31] in KC eyes will change, and thus alter the eyes’ biomechanical properties. Some studies have suggested that the local reduction of corneal biomechanical properties leads to the thinning and softening of some areas of the cornea, following which, KC occurs [32]; the biomechanical properties of the cornea are of great significance for detecting subclinical KC [25]. In this study, CCT was controlled, and no differences were detected in age among the three groups. The differences of A1DA among the three groups may be caused by the changes in corneal biomechanical properties (A1DA and HCDA have been shown to increase with age [11]).

A previous study showed that the resistance of keratoconic corneas to deformation was lower than that of the normal cornea [25]. Research has found that the values of SP-A1 and HCR in KC were lower than those in the normal cornea [12, 15, 28], and HCR could be used to distinguish among KC, subclinical KC, and normal corneas [33]. The CBI can distinguish KC from normal thickness cornea (cutoff > 0.5) [34], and CBI > 0.5 indicates a high risk of developing ectasia [34], especially in cases when the tomographic examinations do not show any abnormality [9, 35]. In addition, one report demonstrated that a parameter related to corneal stiffness could be used as a reliable index to distinguish KC from normal eyes [36]. It should be noted that in the above studies, the CCT of KC and subclinical KC were thinner compared with normal corneas. In this study, we did not detect any statistical difference in SP-A1 between FFKC and TNC groups, MKC and TNC groups, and CBI had lower strength (AUC = 0.624) in differentiating MKC from TNC. In addition, CBI > 0.5 in the TNC group suggests that CBI could not be used to differentiate FFKC and MKC from TNC when TCT ≤ 500 µm. The SP-A1 value is lower in thinner corneas than that in normal corneas [13, 37]. Notably, the control group in our study was TNC (mean value of CCT was about 490 μm), and the corneal thickness was significantly lower than that of the normal cornea (mean value of CCT was about 550 μm), which may be the result of low SP-A1 value of TNC. This phenomenon suggests that results of Corvis ST should be carefully analyzed when SP-A1 is used to distinguish KC from TNC when the TCT is ≤ 500 µm.

The current results show that the DA of the MKC group is greater than that of the TNC group (*P* < 0.05) albeit with a low sensitivity (44.0%) for differentiating MKC from TNC. It was found that when CCT and IOP were controlled, the DA of KC was higher than that of TNC, but, there was no ideal cut-off value [38]. In this study, we excluded the influence of CCT on DCR parameters, and no significant differences were detected for age and bIOP among the three groups (*P* > 0.05). Therefore, the difference of DCR parameters between FFKC and TNC, MKC and TNC may be attributed to the corneal biomechanical properties caused by the change in the corneal microstructure but needs further support from the pathology of FFKC and MKC.

Keratoconus diagnosis model combining DCR and corneal tomographic parameters by BP neural network showed a more accurate diagnostic efficiency was about 91.1%. To improve the accuracy and efficiency of early or mild KC diagnosis, some researchers used machine learning to diagnose keratoconus [39, 40]. Zou et al. [39] based on the 27 parameters of normal cornea, subclinical keratoconus, and KC output using the Pentacam, the accuracy of the diagnostic model constructed by machine learning was as high as 95%. Ruiz et al. [40] constructed the machine learning diagnostic model with 25 Pentacam parameters, for which the accuracy for distinguishing between KC and normal cornea was 93%, and that of subclinical keratoconus and normal cornea was 65%. In this study, when the CCT among TNC, FFKC and MKC was matched, we established a keratoconus diagnosis model which used 13 parameters given by Corvis ST and Pentacam. The accuracy of the BP model is 91%, of which the accuracy of MKC and TNC is 96.7%, and the accuracy of FFKC and TNC is 90%. Although the accuracy of our diagnostic model is slightly lower than that of Zou et al. [39], it should be noted that the parameters of the diagnostic model constructed in the above study are all from Pentacam, and there is no grading of KC, which may make the accuracy of KC constructed in the above study higher. This shows that the biomechanical properties of the cornea play an important role in the diagnosis of early KC. Since the change in biomechanics is earlier than morphological changes in KC [30], the construction of a diagnostic model based on Corvis ST and Pentacam parameters may be more conducive to the discovery of early KC. Therefore, the diagnostic model of KC constructed by us may be more suitable for KC diagnosis when the corneal thickness is thin.

Nevertheless, the present study has some limitations. The maintenance of the cornea depends on the corneal biomechanical properties. Herein, we compared the corneal biomechanical and topography parameters in FFKC and MKC groups, however, the relationship between DCR parameters and topographic map parameters was not explored. Thus, we can further explore the correlation between these parameters to provide guidance for the detection of KC. In this study, the number of patients included was limited, and hence, should be expanded to substantiate these observations.

## Conclusions

When CCT among TNC, FFKC, and MKC are matched, the majority of the tomographic and DCR parameters were different among the three groups. IHD and partial DCR parameters from Corvis ST showed that FFKC and MKC could be distinguished from TNC.

## Data Availability

The data used to support the findings of this study are available from the corresponding author upon request.
